# Lateral Fluid Percussion Injury Impairs Hippocampal Synaptic Soluble N-Ethylmaleimide Sensitive Factor Attachment Protein Receptor Complex Formation

**DOI:** 10.3389/fneur.2017.00532

**Published:** 2017-10-10

**Authors:** Shaun W. Carlson, Jeremy Henchir, C. Edward Dixon

**Affiliations:** ^1^Department of Neurosurgery, Safar Center for Resuscitation Research, University of Pittsburgh, Pittsburgh, PA, United States; ^2^V.A. Pittsburgh Healthcare System, Pittsburgh, PA, United States

**Keywords:** sensitive factor attachment protein receptor, α-synuclein, traumatic brain injury, synapse, neurobehavioral function

## Abstract

Traumatic brain injury (TBI) and the activation of secondary injury mechanisms have been linked to impaired cognitive function, which, as observed in TBI patients and animal models, can persist for months and years following the initial injury. Impairments in neurotransmission have been well documented in experimental models of TBI, but the mechanisms underlying this dysfunction are poorly understood. Formation of the soluble N-ethylmaleimide-sensitive factor attachment protein receptor (SNARE) complex facilitates vesicular docking and neurotransmitter release in the synaptic cleft. Published studies highlight a direct link between reduced SNARE complex formation and impairments in neurotransmitter release. While alterations in the SNARE complex have been described following severe focal TBI, it is not known if deficits in SNARE complex formation manifest in a model with reduced severity. We hypothesized that lateral fluid percussion injury (lFPI) reduces the abundance of SNARE proteins, impairs SNARE complex formation, and contributes to impaired neurobehavioral function. To this end, rats were subjected to lFPI or sham injury and tested for acute motor performance and cognitive function at 3 weeks post-injury. lFPI resulted in motor impairment between 1 and 5 days post-injury. Spatial acquisition and spatial memory, as assessed by the Morris water maze, were significantly impaired at 3 weeks after lFPI. To examine the effect of lFPI on synaptic SNARE complex formation in the injured hippocampus, a separate cohort of rats was generated and brains processed to evaluate hippocampal synaptosomal-enriched lysates at 1 week post-injury. lFPI resulted in a significant reduction in multiple monomeric SNARE proteins, including VAMP2, and α-synuclein, and SNARE complex abundance. The findings in this study are consistent with our previously published observations suggesting that impairments in hippocampal SNARE complex formation may contribute to neurobehavioral dysfunction associated with TBI.

## Introduction

The connection between traumatic brain injury (TBI) and cognitive dysfunction has been well described in patients afflicted with a TBI, and in multiple experimental models with fidelity to the pathologies observed in clinical TBI. TBI patients describe problems in memory, attention, limitations at work, and learning for years following the injury, that directly contribute to reduced quality of life (1–5). Damage to the hippocampus, visualized by imaging and in postmortem studies, may directly contribute to the manifestation of the cognitive impairments (6–9).

Previous work in multiple experimental models of TBI highlights that the primary or secondary mechanisms of injury disrupt synaptic function (10–15). Prior studies demonstrate that multiple experimental TBI models with features of focal and/or diffuse injury impair neurotransmission in multiple brain regions, including the hippocampus, cortex, and striatum (11, 15–17). Scheff et al. (18) showed that TBI produced by controlled cortical impact (CCI) results in the loss of hippocampal synapses; however, recent ultrastructural findings suggest that changes within surviving synapses after TBI can also contribute to dysfunction (19). While published studies evaluated changes after CCI in neurotransmitter-specific transporters, receptors, and enzymes for synthesis as contributing to impaired neurotransmission (20–24), neurotransmission deficits, in multiple brain regions with varying proximity to the site of injury, suggest that TBI may impair conserved machinery within synapses throughout the brain.

In the synapse, the release of neurotransmitters from the vesicle is facilitated by the formation of the *N*-ethylmaleimide–sensitive factor attachment protein receptor (SNARE) complex. The formation of the SNARE complex is an important step in initiating vesicular docking and fusion at the plasma membrane (25, 26). Multiple proteins are associated with and comprise the SNARE complex, including cysteine string protein α (CSPα), α-synuclein (α-syn), synaptobrevin2 (VAMP2), syntaxin-1, and synaptosomal-associated protein of 25 kDa (SNAP-25). Published work employing targeted genetic manipulations highlights the important role these proteins play in the regulated formation and abundance of the SNARE complex (27–31), and that reductions in monomeric proteins disrupt SNARE complex assembly. We recently showed that CCI reduces the abundance of multiple key SNARE proteins and SNARE complexes in the weeks following CCI injury (19, 32). One potential confound of these studies was the extent of neuronal and synaptic loss following CCI, making it difficult to delineate the magnitude of SNARE protein alterations from tissue loss. To address this, we employed the lateral fluid percussion injury (lFPI) model to evaluate a less severe histopathological injury with reduced cortical and hippocampal loss.

In this study, we sought to evaluate the effect of lFPI on synaptic abundance of SNARE proteins and complexes. To focus the evaluation of SNARE proteins in the injured synapse, hippocampal tissues were processed to yield synaptosomal-enriched lysates. The abundance of SNARE proteins and SNARE complexes was assessed at 1 week post-injury, as this time point has been associated with reduced SNARE proteins in the hippocampus following CCI (19). lFPI resulted in a significant reduction in α-syn, VAMP2, and SNAP-25 complexes. The lFPI significantly impaired acute motor performance and cognitive function at 3 weeks post-injury. The current findings highlight that lFPI alters the intrasynaptic abundance of SNARE proteins and complexes and may contribute to neurobehavioral dysfunction after TBI.

## Materials and Methods

### Animals

All experimental procedures were approved by the University of Pittsburgh Institutional Animal Care and Use Committee in accordance with the guidelines established by the National Institutes of Health in the Guide for the Care and Use of Laboratory Animals. Animals were housed up to two rats per cage in the University of Pittsburgh vivarium with a 12:12 light/dark photoperiod (light on at 7:00 a.m.) and provided food and water *ad libitum*. Adult male Sprague-Dawley rats (Harlan, Indianapolis, IN, USA) weighing 275–375 g were utilized in this study.

### Fluid Percussion Injury

Animals were subjected to lFPI as previously described (33, 34). Rats were anesthetized using 4% isoflurane in a 2:1 N_2_O/O_2_ mixture within a ventilated anesthesia chamber. Following endotracheal intubation, rats were mechanically ventilated and anesthesia maintained with 2% isoflurane mixture. Animals were placed in a stereotaxic frame in the supine position, and body temperature was monitored by rectal thermistor probe and maintained at 37°C using a heating pad. Following a midline incision, the soft tissues were reflected. A craniotomy was completed, using a 4.7 mm trephine, between bregma and lambda, centered 5 mm lateral of the sagittal suture to expose the dura mater over the right parietal cortex. Set screws were secured in pilot holes at least 5 mm from the craniotomy. A Luer-lock hub was fitted to the craniotomy and secured with cyanoacrylate gel to adhere and seal the hub to the skull. The hub was secured to the set screws by application of methyl-methacrylate (Henry Schein, Melville, NY, USA). Once the methyl methacrylate was hardened, the hub was filled with sterile saline and connected to the injury device. Animals were randomly assigned to receive either sham or lFPI. Sham animals were subjected to all surgical procedures except the induction of the injury. For induction of the lFPI, the pendulum hammer was released onto the piston of the fluid filled cylinder to induce the injury (2.0 atm). The injury hub was removed, and the head sutured. Apnea time and righting reflex time were recorded for each animal. Once ambulatory, the animals were returned to their home cage.

### Vestibular Motor Function

A total of 21 rats (*n* = 8 sham and *n* = 13 lFPI) were utilized to examine vestibulomotor function, as previously described (35). Assessments of beam balance and beam walking were completed on days 1–5 post-injury with training completed on day 0 before injury. Gross vestibulomotor function was evaluated with a beam balance task. The animal was placed on a suspended, narrow wooden beam (1.5 cm width) 30″ above a padded surface. The latency on the beam, maximum of 60 s, was measured. Each animal was tested over three trials per day with a 30 s rest period between trials. A modified beam walking task evaluated finer components of vestibulomotor performance. Before injury, the animal was trained to escape a loud white noise and bright light by traversing the narrow wooden beam (2.5 cm × 100 cm) to enter a darkened goal box at the opposite end. Four pegs (4 cm high and 3 mm in diameter) were spaced equally along the center of the beam to increase task difficulty. If the rats fell off the beam or did not transverse the beam in the allotted 60 s, the noise and light were stopped, and the rat was placed in the goal box. The average latency to traverse the beam for three trials per day was measured.

### Spatial Learning

The same cohort of rats subjected to vestibulomotor testing was evaluated for spatial learning and memory. Spatial learning was evaluated using the Morris water maze (MWM) task using a video-tracking system (AnyMaze, Stoelting, Inc., Wood Dale, IL, USA) as previously described (35). A circular tank (180 cm diameter and 45 cm high) was filled with 26 ± 1°C water to a height of 30 cm to conceal a transparent circular platform (10 cm in diameter and 29 cm high) located in a fixed position 45 cm from the wall. Visual cues outside of the maze aid the animal in locating the escape platform. Testing began on day 14 post-injury, without previous training or exposure to the MWM. Testing continued for five consecutive days (14–18 days post-injury) with each animal completing four trials per day. Rats were randomly placed in the water against the tank wall and released to freely swim in the tank to find the hidden platform within 120 s. If the animal was unable to locate the platform within the allotted time, it was manually directed to the platform. The animal remained on the platform for 30 s and placed into an incubator between trials. Following a 4 min intertrial interval, the next trial was initiated. On day 19 post-injury, the animal was tested using a probe trial paradigm in which the hidden platform was removed. The location of the hidden platform was designated as the target quadrant. A visible platform assessment was completed on days 19 and 20.

### Histology

Rats received an overdose of sodium pentobarbital (intraperitoneally, 100 mg/kg) at 21 days post-injury, following assessment of cognitive function. Animals were transcardially perfused with saline, followed by a mixture of 10% neutral-buffered formalin (Fisher Scientific, Waltham, MA, USA). The brains were post-fixed for 24 h in fixative and cryoprotected with 30% sucrose in 0.1 M phosphate-buffered saline. The brains were frozen and cut into 35 µm thick coronal sections with a cryostat (Leica Microsystems Inc., Buffalo Grove, IL, USA). A cohort of 12 (*n* = 6 per group) was randomly selected to evaluate regional cell loss. Sections from each animal were stained with cresyl violet at every 1 mm. Sections between bregma −2.5 and −5.0 mm were utilized for immunohistochemical analysis. Sections were treated with 0.9% H_2_O_2_ in 50% methanol to block endogenous peroxidases and washed with TBS. Sections were blocked with 5% normal donkey serum in TBS with 0.1% triton X-100 and incubated overnight in anti-NeuN primary antibody (Millipore, Billerica, MA, USA). Following incubation of directly conjugated horse radish peroxidase secondary antibody, sections were washed, and the reaction product was visualized using DAB as a substrate. Cresyl violet and NeuN stained sections were imaged with a Nikon 90i (Melville, NY, USA) to examine overt cortical and hippocampal cell loss.

### Tissue Preparation for Western Blot Analysis

A cohort of 12 rats (*n* = 6 per group) was subjected to injury as described earlier. At 7 days post-injury, animals received an overdose of sodium pentobarbital, and the brains were removed. The ipsilateral hippocampus was rapidly dissected on a chilled ice plate, immediately snap frozen in liquid nitrogen and stored at −80°C. Hippocampal samples were processed to produce synaptosomal-enriched lysates. The tissues were homogenized using a dounce homogenizer with SYN-PER isolation reagent (Thermo Scientific, Pittsburgh, PA, USA) with protease inhibitors (Sigma-Aldrich, St. Louis, MO, USA). The homogenized lysates were centrifuged at 1,200 × *g* at 4°C for 10 min, supernatants collected, and centrifuged at 15,000 × *g* at 4°C for 20 min. The pellet was resuspended in isolation buffer and protein concentration determined by a BCA protein assay kit (Thermo Scientific) using a 96-well microplate reader (Biotek, Winooski, VT, USA).

### Immunoblotting

To examine monomeric SNARE proteins in the hippocampus, synaptosomal lysates were boiled for 10 min before SDS-PAGE. SNARE complex abundance was assessed in unboiled lysates to retain high-molecular weight complexes, and were defined as SNAP-25 immunoreactive material between 50 and 250 kDa that was absent from boiled samples, as previously described (19, 28, 32). Lysates (20 µg/well) and molecular weight markers were separated by SDS-PAGE. The proteins were electrophoretically transferred to a PVDF membrane (Invitrogen, Carlsbad, CA, USA). Membranes were incubated in 5% nonfat dry milk in tris-buffered saline containing 0.1% Tween 20 (blocking solution) for 1 h. Primary antibodies were diluted in blocking solution and incubated overnight at 4°C. The primary antibodies utilized were as follows: rabbit anti-CSPα (Thermo-Fisher, Rockford, IL, USA), mouse anti-SNAP-25 (Biolegend, San Diego, CA, USA), mouse anti-syntaxin-1 (Sigma-Aldrich), rabbit anti-VAMP2 (Millipore, Temecula, CA, USA), mouse anti-α-syn (BD Transduction, San Jose, CA, USA), rabbit anti-synaptophysin (Abcam, Cambridge, MA, USA), and mouse anti-β-actin (Sigma-Aldrich). On the following day, the membranes were washed with 1× TBS buffer, incubated in blocking solution containing horseradish peroxidase-conjugated secondary antibodies for 1 h. Proteins were visualized with a chemiluminescence detection system (Supersignal, Pierce) and imaged with a Chemidoc Imager (BioRad). To normalize for protein content, the membranes were stripped and reprobed with mouse anti-β actin antibody (Sigma-Aldrich). Sham and lFPI lysates were loaded in the same gel for comparison. Optical density of each protein of interest was measured using ImageJ (National Institutes of Health). The optical density of β-actin was measured for each lane and used for normalization as a control for each sample. Values are presented as the ratio of optical densities of samples as a percentage of sham. Data are expressed as the group means ± SEM.

### Statistical Analyses

Testing and quantification were completed by investigators blinded to the injury condition of each animal. Data are presented as mean ± SEM. Statistical comparisons of beam balance latency, beam walking latency, and spatial acquisition were completed using a two-way analysis of variance followed by Tukey’s *post hoc* analyses, when appropriate. Probe trial performance was compared using a Student’s *t*-test. Immunoblot data were compared using a Student’s *t*-test for each protein of interest. The *p* values reported in the text are the results of the *post hoc* analyses, unless stated. Statistical tests were completed using GraphPad Prism (GraphPad, La Jolla, CA, USA). A *p* value less than 0.05 was considered statistically significant for all tests.

## Results

### lFPI Results in Motor and Cognitive Impairments and Regional Cell Loss

**Figure 1:**
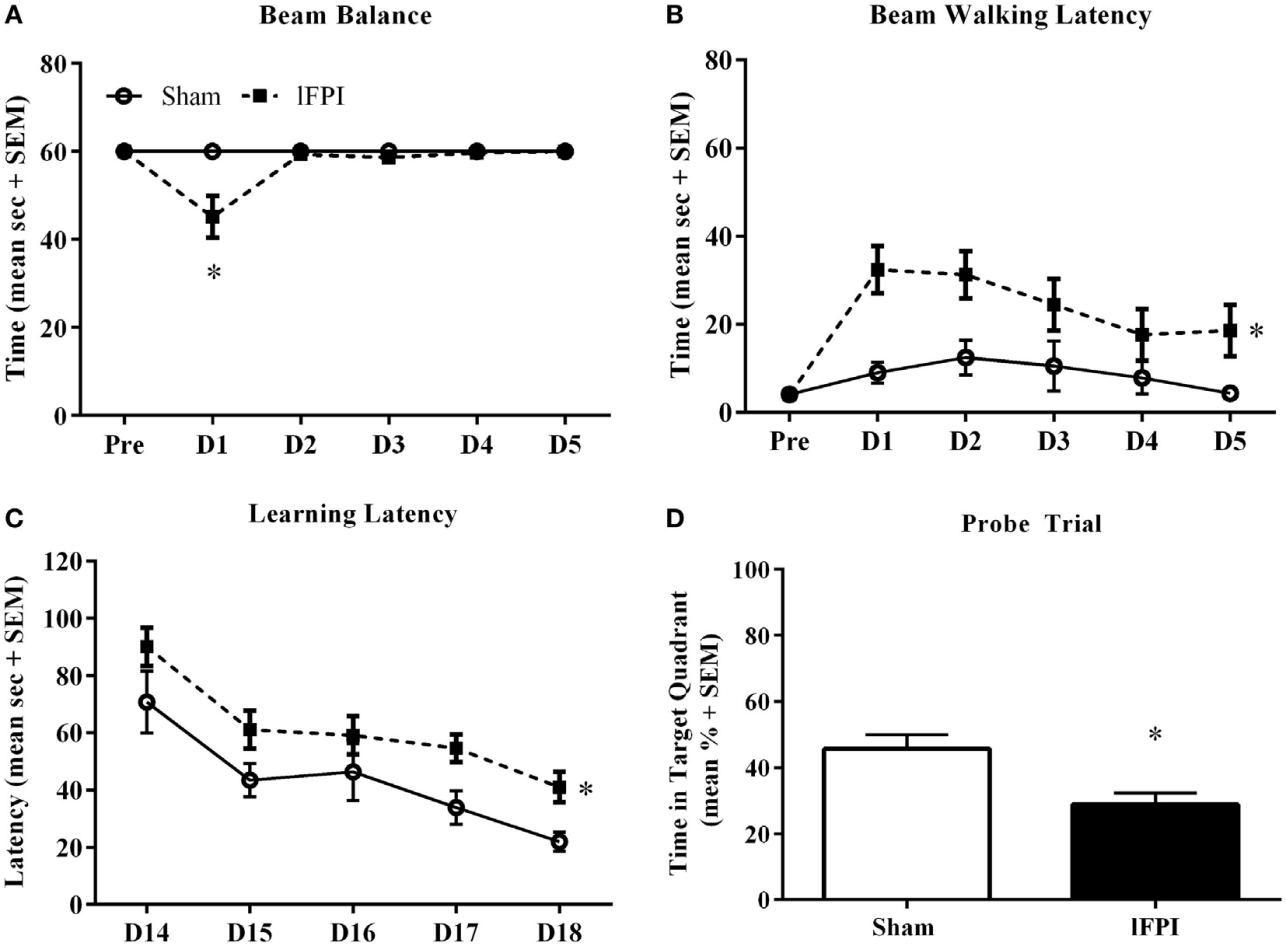
Lateral fluid percussion injury (lFPI) impairs motor and cognitive function. **(A)** Assessment of vestibulomotor function using the beam balance and beam walking tasks on days 1–5 post-injury, revealed lFPI produced a transient beam balance deficit at 1 day post-injury, compared with sham injury (*p < 0.01). **(B)** lFPI-injured animals exhibited a significant increase in beam walking latency on days 1–5 post-injury compared with sham injury (*p < 0.01). **(C)** Assessment of spatial acquisition over days 14–18 post-injury, revealed a significant increase in latency time to platform in animals subjected to lFPI, compared with sham injury (*p < 0.001). **(D)** In the probe trial task, lFPI-injured animals exhibited a significant reduction in time in the target quadrant compared with sham-injured animals (*p < 0.01) (*n* = 8 sham and *n* = 13 lFPI).

Beam balance and beam walking tasks were completed to evaluate acute vestibulomotor dysfunction following lFPI. In the beam balance task, animals subjected to lFPI exhibited a transient reduction in balance duration at 1 day post-injury (45.2 ± 0.6 s), compared with sham (60.0 ± 0 s; *post hoc t*-test *p* < 0.01; injury effect *p* < 0.01, time effect *p* < 0.01, interaction *p* < 0.01; Figures 1A). Analysis of beam walking performance revealed a significant increase in latency times of lFPI-injured rats over the duration of testing, compared with sham rats (injury effect, *p* < 0.01, time effect *p* < 0.01; Figure 1B).

Assessment of cognitive performance was completed using the Morris water maze task beginning on day 14 post-injury. Animals subjected to lFPI exhibited a significant increase in latency times over the testing period, compared with sham animals (injury effect; *p* < 0.001, time effect *p* < 0.001; Figure 1C). In the hidden platform probe trial, lFPI-injured animals (28.9 ± 3.5%) showed significantly reduced time in the target quadrant compared with sham animals (45.6 ± 4.5%; *p* < 0.01; Figure 1D).

**Figure 2:**
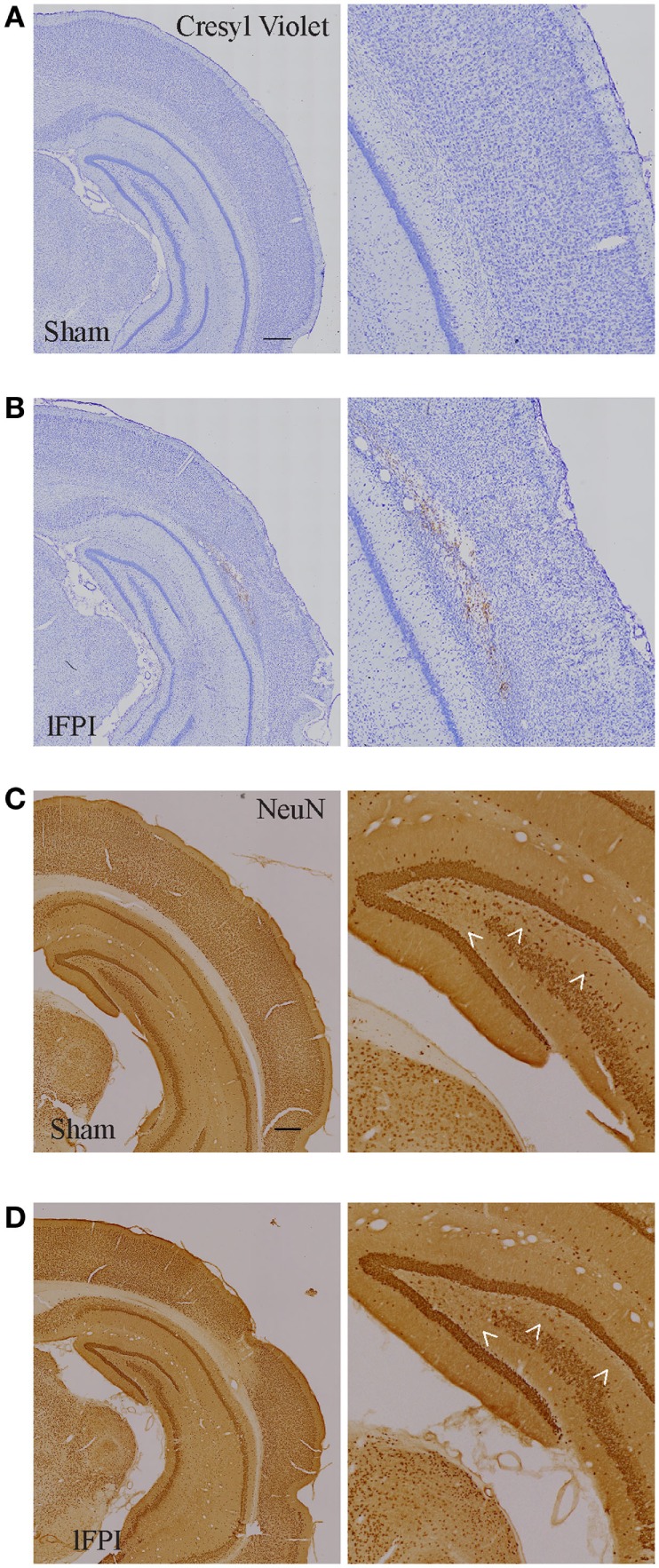
Lateral fluid percussion injury (lFPI) results in cortical damage and hippocampal neuron loss. **(A,B)** Representative images of cresyl violet stained and **(C,D)** NeuN-immunohistochemical stained coronal brain sections after sham and lFPI. lFPI produced a lesion in the cortex at the gray and white matter interface and an appearance of hilar neuron loss (arrowheads) at 3 weeks post-injury (scale bar = 500 µm, *n* = 6 sham and *n* = 6 lFPI).

Qualitative histological evaluation of the cortex and hippocampus with cresyl violet staining and NeuN immunohistochemistry showed the formation of a cortical contusion at 3 weeks following lFPI, in agreement with previous reports (35, 36). Consistent with these reports, lFPI produced cortical tissue necrosis, gliosis, and hemorrhage at the interface between the neocortex and white matter (Figures 2A,B), which were not observed in animals subjected to a sham surgery. Qualitative assessment of immunohistochemical staining of NeuN revealed an apparent loss of hippocampal hilar neurons after lFPI (Figures 2C,D).

### lFPI Reduces the Abundance of Synaptic α-Syn in Hippocampal Synapses

**Figure 3:**
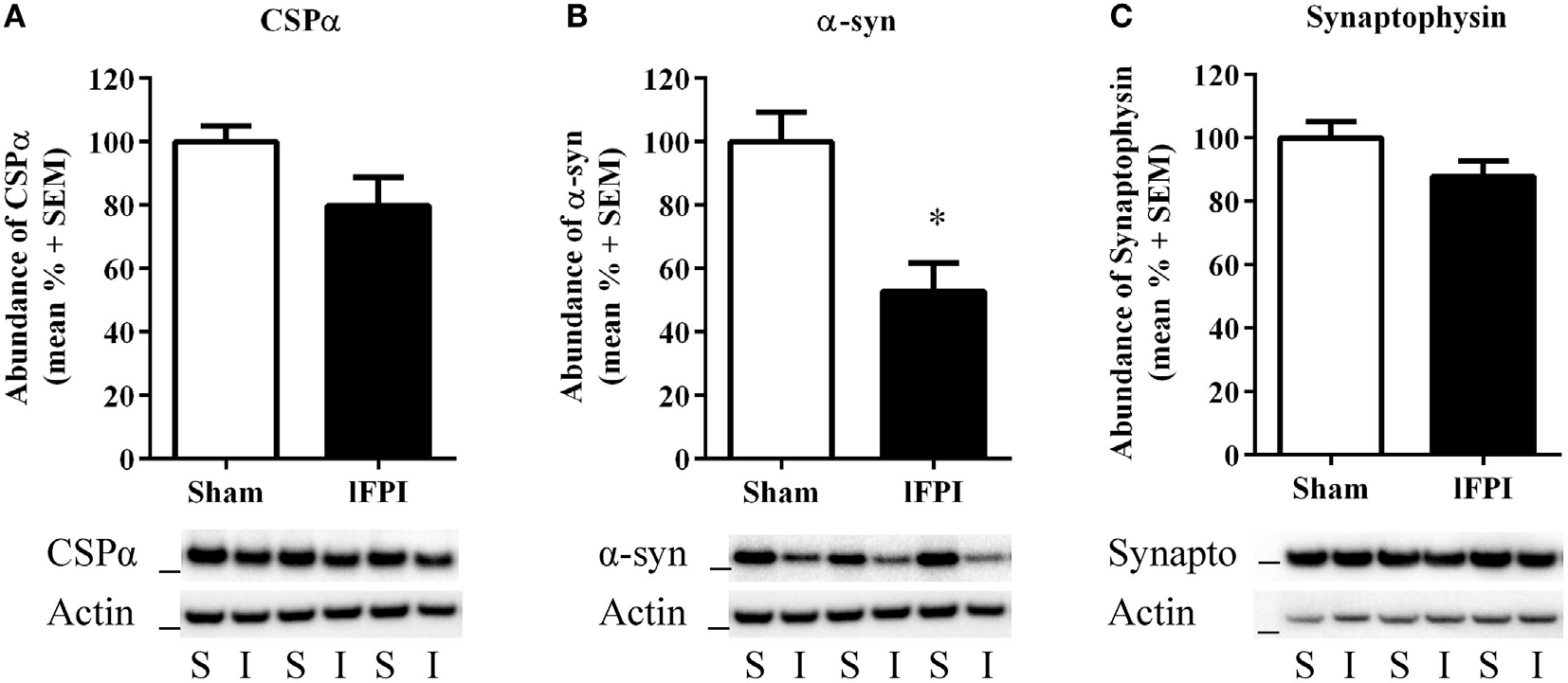
Lateral fluid percussion injury (lFPI) reduced α-syn abundance in the hippocampus. **(A)** Representative western blot image of CSPα in hippocampal synaptosomal lysates at 1 week post-injury (*n* = 3 per group shown). Assessment of CSPα (34 kDa, marker is 28 kDa) in lFPI-injured rats (I) revealed a trend toward reduced abundance compared with sham (S) rats, but it did not reach significance. **(B)** Representative western blot image of α-syn (18 kDa, marker is 14 kDa) in lFPI-injured rats revealed a significant reduction compared with sham rats (*p < 0.005). **(C)** Representative western blot image of synaptophysin (35 kDa, marker is 28 kDa) in lFPI-injured rats revealed a small reduction in abundance compared with sham rats, but it did not reach significance. Proteins were normalized to actin (42 kDa, marker is 38 kDa) (*n* = 6 sham and *n* = 6 lFPI).

We next sought to examine the effect of lFPI on SNARE proteins in hippocampal synapses. Semi-quantitative immunoblotting of hippocampal synaptosomal-enriched lysates was completed to evaluate the abundance of the chaperone SNARE protein CSPα, α-syn, and synaptophysin at 1 week post-injury. Tissue from rats subjected to lFPI exhibited a 20.2 ± 9.0% reduction in CSPα abundance in hippocampal synapses as compared with sham surgery; however, it did not reach significance (Figure 3A). Assessment of α-syn revealed rats subjected to lFPI showed a 47.2 ± 9.0% reduction compared with rats subjected to sham surgery (*p* < 0.005; Figure 3B). The level of synaptophysin in lFPI-injured rats was reduced by 12.2 ± 4.9% and was not significantly different from sham-injured rats (Figure 3C).

### lFPI Reduces the Abundance of VAMP2 in Hippocampal Synapses

**Figure 4:**
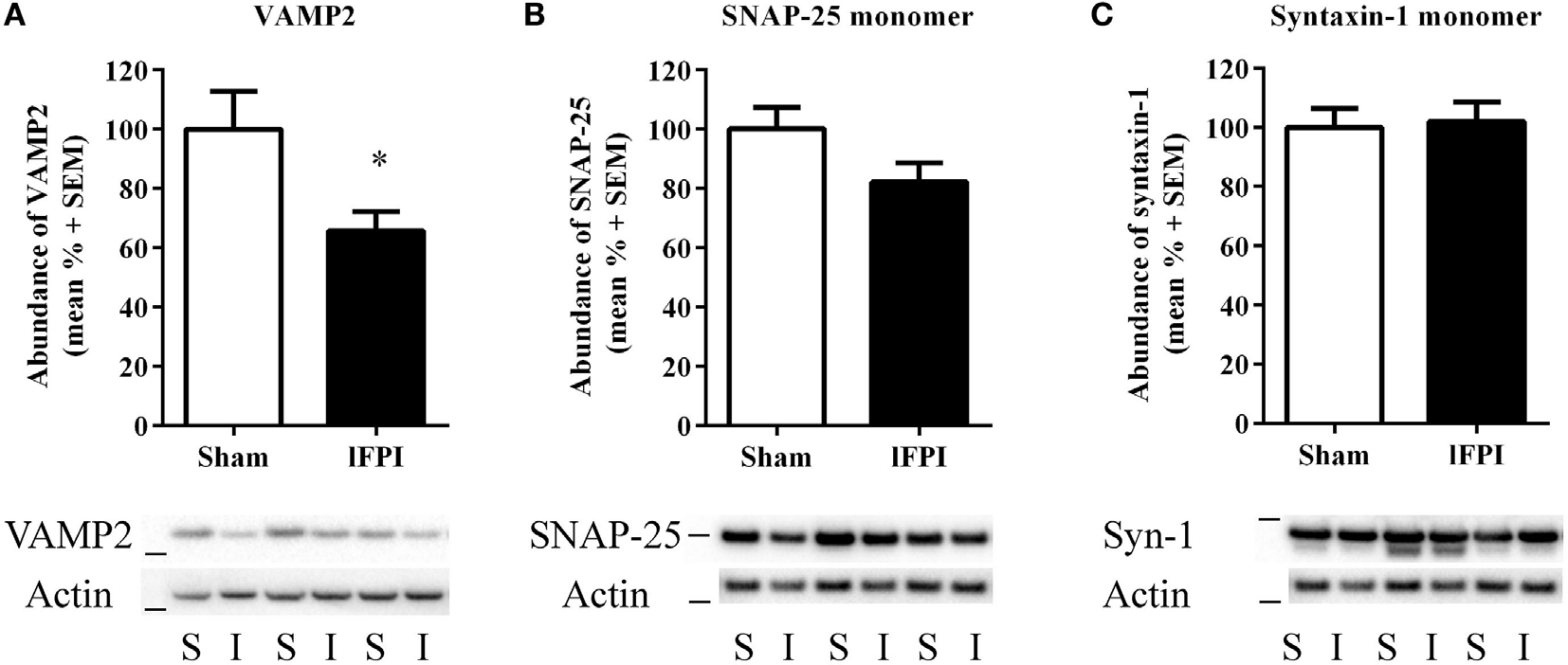
Lateral fluid percussion injury (lFPI) reduced VAMP2 abundance in the hippocampus. **(A)** Representative western blot image of VAMP2 in hippocampal synaptosomal lysates at 1 week post-injury (*n* = 3 per group shown). Assessment of VAMP2 (18 kDa, marker is 14 kDa) in lFPI-injured rats (I) revealed significantly reduced abundance compared with sham (S) rats (*p < 0.01). **(B)** Representative western blot image of synaptosomal-associated protein of 25 kDa (SNAP-25) monomer (25 kDa, marker is 28 kDa) in lFPI-injured rats revealed a modest reduction compared with sham rats, but it did not reach significance. **(C)** Representative western blot image of syntaxin-1 monomer (35 kDa, marker is 38 kDa) in lFPI-injured rats revealed no change in abundance compared with sham rats. Proteins were normalized to actin (42 kDa, marker is 38 kDa) (*n* = 6 sham and *n* = 6 lFPI).

Evaluation of VAMP2, a SNARE protein located on vesicular membranes, was significantly reduced by 34.3 ± 6.5% in lFPI-injury rats compared with sham-injured rats (*p* < 0.05) at 1 week post-injury (Figure 4A). Evaluation of the monomeric SNAP-25 and syntaxin-1 proteins revealed no significant reduction in either protein after lFPI as compared with sham abundance (17.83 ± 6.4 and 2.0 ± 6.7%, respectively; Figures 4B,C).

### lFPI Reduces SNARE Complex Abundance in Hippocampal Synapses

**Figure 5:**
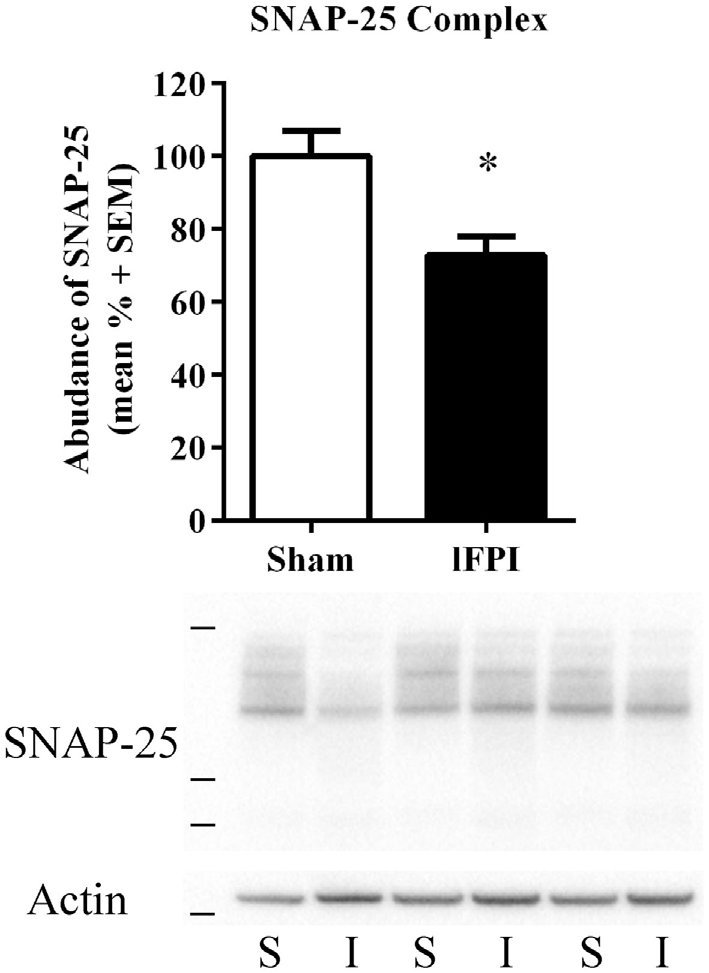
Lateral fluid percussion injury (lFPI) reduced soluble N-ethylmaleimide sensitive factor attachment protein receptor complex formation in the hippocampus (SNARE). Representative western blot image of synaptosomal-associated protein of 25 kDa (SNAP-25) immunoreactive SNARE complexes in hippocampal synaptosomal lysates at 1 week post-injury (*n* = 3 per group shown). Assessment of high-molecular weight SNARE complexes (markers are 38, 49, and 198 kDa) in lFPI-injured rats (I) revealed significantly reduced abundance compared with sham (S) rats (*p < 0.5) SNAP-25 complexes were normalized to actin (42 kDa, marker is 38 kDa) (*n* = 6 sham and *n* = 6 lFPI).

Finally, we sought to evaluate the effect of lFPI on SNARE complex formation by assessing high-molecular weight SNAP-25 immunoreactivity in unboiled synaptosomal-enriched lysates, as observed by immunoreactive bands between 50 and 250 kDa. SNAP-25 complex abundance was significantly reduced in rats subjected to lFPI (27.2 ± 5.1%) as compared with rats subjected to sham surgery at 1 week post-injury (*p* < 0.05; Figure 5).

## Discussion

The objective of this study was to examine the effect of lFPI injury on the hippocampal abundance of the SNARE proteins critical for vesicular docking and fusion in the synapse. We provide novel evidence of lFPI-induced reductions in the abundance of monomeric SNARE proteins and SNARE complex formation in hippocampal synapses. The severity of injury utilized in this study resulted in neurobehavioral impairments and qualitative histopathological changes that have been well described for the model of lFPI. We previously published that CCI significantly reduces the abundance of SNARE proteins and complexes (19, 32); however, it was unknown if this pathological response is present in models of reduced overt neuropathology. The current findings provide evidence that reductions in SNARE proteins and SNARE complex formation occur following a moderate lFPI, and taken together with our previously published findings, SNARE alterations have been described in two models of experimental TBI. These data suggest that alterations in synaptic SNARE proteins may be a contributing mechanism to synaptic dysfunction across the range of TBI severities.

The FPI injury model is a well characterized and widely utilized experimental model of TBI, originally developed for use in the rabbit and cat (37, 38), and subsequently adapted for mice and rats (33, 34, 39–43). The development of neurobehavioral dysfunction following lFPI replicates the motor and cognitive impairments clinically reported by individuals afflicted with a TBI (3, 44). This study found impaired motor function, as assessed by beam walking and beam balance task performance during the week post-injury, consistent with previously reported observations (3, 33, 34, 45, 46). Assessment of cognitive dysfunction with the Morris water maze task showed increased latency during spatial acquisition and reduced probe trial performance at 3 weeks post-injury. Visual inspection of cresyl violet and NeuN as indicators of overt cell and neuronal loss showed cortical and hippocampal damage that appeared consistent with previous measurements of cell loss, including stereological quantification, following moderate lFPI (34, 36, 40, 47, 48); however, the extend of cell loss was not quantified. The findings in this study highlight neurobehavioral dysfunction and histopathology commonly associated with moderate lFPI.

Assembly of the SNARE complex is a key step in the release of neurotransmitters from the synaptic vesicle (25, 26). Published reports detail that loss of individual monomeric SNARE proteins, including CSPα, VAMP2, and SNAP-25, impair SNARE complex formation and reduce vesicle docking (27–31, 49). *In vitro* findings utilizing cultured neurons from VAMP2 knockout mice exhibit reduced synaptic vesicular docking during hypertonic sucrose and Ca^2+^-triggered fusion (49). CSPα knockout mice exhibit reduced SNAP-25 abundance and SNARE complex formation (29). Triple transgenic synuclein knockout mice display impaired SNARE complex formation, which is restored following α-synuclein supplementation (27). Together, these mechanistic studies highlight the connection between monomeric SNARE protein loss and impaired SNARE complex assembly.

We previously showed that the severe CCI model of TBI reduces several monomeric SNARE proteins and impairs the formation of the SNARE complex in the days and weeks following TBI (19, 32). lFPI resulted in a robust reduction in α-syn and VAMP2 at 1 week post-injury. The abundance of SNAP-25 immunoreactive SNARE complexes was significantly reduced at 1 week post-injury. The reduction in monomeric SNARE proteins and SNARE complex formation in this study appeared less robust than our previous findings following CCI (19, 32); however, it is difficult to extrapolate the severity-dependent decreases in SNARE protein abundance between these two models. The reductions in key SNARE proteins and SNARE complexes after lFPI were not dependent on overt changes in synaptic density, as assessed by synaptophysin, in synaptosomal-enriched lysates. Future studies will need to investigate the time course of SNARE complex assembly impairments in multiple regions of the brain following lFPI.

Previous reports have described hippocampal dysfunction following midline and lateral FPI. Tonic levels of glutamate in the dentate gyrus are elevated at 2 days after midline FPI, but potassium-evoked glutamate release in the dentate gyrus was not altered (11). Electrophysiological recordings in the injured hippocampus following FPI revealed pathological alterations in long-term potentiation in the weeks post-injury (50–52). The electrophysiological changes in the dentate gyrus after FPI have been attributed to a loss of inhibition and increased hyperexcitability, and a lower seizure threshold (53–56). Additional work is needed to understand if FPI results in reduced SNARE complex formation in excitatory or inhibitory synapses, or if the reduction occurs independent of neuronal type. Ultrastructural electron micrographs of hippocampal synapses at 1 week following CCI show reduced vesicle density and altered vesicular distribution that could contribute to impaired neurotransmitter release (19); however, future work will need to determine if similar intrasynaptic vesicular changes occur following FPI. It is possible that intrasynaptic impairments in SNARE complex formation contribute to the alterations in hippocampal neurotransmitter release and electrophysiological properties reported in the weeks following TBI.

In summary, we found novel evidence of intrasynaptic protein changes that may directly contribute to the development of neurotransmission impairments and neurobehavioral dysfunction following TBI. This is the first study to evaluate the SNARE protein machinery, important for vesicle docking and fusion, in the synapse following lFPI. We observed a significant reduction in key monomeric SNARE proteins and reduced SNARE complex formation in hippocampal synaptosomes at 1 week after lFPI. Together with our previous findings, this work provides evidence of intrasynaptic changes in SNARE complex formation in two models of experimental TBI. This study provides a new line of evidence that intrasynaptic alterations in the SNARE complex may contribute to impaired neurotransmission and behavioral dysfunction after TBI.

## Ethics Statement

All experimental procedures were approved by the University of Pittsburgh Institutional Animal Care and Use Committee in accordance with the guidelines established by the National Institutes of Health in the Guide for the Care and Use of Laboratory Animals.

## Author Contributions

SC, JH, and CD designed and performed the experiments and analyzed data. SC and CD wrote the paper.

## Conflict of Interest Statement

The authors declare that the research was conducted in the absence of any commercial or financial relationships that could be construed as a potential conflict of interest.
